# Ossifying Fibromyxoid Tumor of Soft Parts in the Head and Neck: A Systematic Review Addressing Surgical Management and Adjuvant Therapies

**DOI:** 10.3390/cancers17091508

**Published:** 2025-04-29

**Authors:** Gianluca Scalia, Valentina Zagardo, Zubayer Shams, Gianluca Ferini, Salvatore Marrone, Eliana Giurato, Francesca Graziano, Giancarlo Ponzo, Massimiliano Giuffrida, Massimo Furnari, Giuseppe Emmanuele Umana, Giovanni Federico Nicoletti

**Affiliations:** 1 Neurosurgery Unit, Department of Head and Neck Surgery, Garibaldi Hospital, 95124 Catania, Italy; gianluca.scalia@outlook.it (G.S.); fgraziano@arnasgaribaldi.it (F.G.); gponzo@arnasgaribaldi.it (G.P.); mgiuffrida@arnasgaribaldi.it (M.G.); mfurnari@arnasgaribaldi.it (M.F.); gnicoletti@arnasgaribaldi.it (G.F.N.); 2 School of Medicine and Surgery, Kore University of Enna, 94100 Enna, Italy; umana.nch@gmail.com; 3 Department of Radiation Oncology, REM Radioterapia srl, 95125 Viagrande, Italy; valentina.zagardo@grupposamed.com; 4 Brunel Medical School, Brunel University London, London UB8 3PH, UK; zubayershams1@gmail.com; 5 Department of Neurosurgery, Sant’ Elia Hospital, 93100 Caltanissetta, Italy; salvo.mr89@gmail.com; 6 Anatomic Pathology Unit, Garibaldi Hospital, 95124 Catania, Italy; egiurato@arnasgaribaldi.it; 7 Department of Neurosurgery, Trauma and Gamma Knife Center, Cannizzaro Hospital, 95126 Catania, Italy

**Keywords:** ossifying fibromyxoid tumor, head and neck, surgical management, adjuvant therapies, histopathological features

## Abstract

**Background:** Ossifying fibromyxoid tumors (OFMTs) are rare mesenchymal neoplasms with behaviors ranging from benign to malignant. Although most occur in the extremities and trunk, 9–13% are found in the head and neck, such as the oral cavity, scalp, and calvarium. Diagnosis is challenging due to their rarity and histological similarity to other neoplasms. This review synthesizes evidence on the clinical presentation, diagnostic features, and treatment outcomes of OFMTs in the head and neck, focusing on surgical management and adjuvant therapies. **Methods:** A systematic review was conducted according to PRISMA guidelines, with searches in PubMed/MEDLINE, Embase, Scopus, and Web of Science. Studies from 1989 to 2024 reporting OFMTs in the head and neck with clinical, histopathological, and treatment data were included. Extracted data encompassed demographics, tumor features, surgical margins, adjuvant therapy, and outcomes. **Results:** Forty studies with 99 patients were included. Patient ages ranged from 3 weeks to 88 years (median 47), with a male predominance (63.64%). The most common presentation was a slow-growing, painless mass. Tumors were most often found in the neck, oral cavity, scalp, and calvarium. Histopathology revealed encapsulated tumors with fibromyxoid stroma, spindle-shaped cells, and a peripheral rim of metaplastic bone in 70% of cases. Immunohistochemistry showed positivity for S-100, vimentin, and SOX10. Surgical excision was the main treatment, used in 28.28% of cases, with recurrence in 9.09% and no metastases. Adjuvant therapies, mainly radiotherapy, were employed in 15.15% of cases. **Conclusions:** OFMTs of the head and neck are rare neoplasms requiring multidisciplinary care. Imaging, histopathology, and immunohistochemistry are key to diagnosis. Surgical excision with clear margins remains the primary treatment, with a low recurrence rate. Adjuvant therapy may be needed for malignant or incompletely excised cases. Further research is needed to optimize follow-up protocols and assess molecular profiling for risk stratification.

## Simple Summary

Ossifying fibromyxoid tumors (OFMTs) are rare soft-tissue tumors that can appear anywhere in the body but are found in the head and neck region in a minority of cases. These tumors often present as slow-growing, painless masses, making diagnosis challenging. This systematic review analyzed 99 cases to better understand how OFMTs in the head and neck are diagnosed and treated. Most tumors showed benign behavior, with low rates of recurrence and no recorded metastasis. Diagnosis relied on imaging, histological analysis, and immunohistochemistry, often showing features like fibromyxoid stroma and bone formation. The primary treatment was complete surgical removal, which usually resulted in excellent outcomes. Adjuvant therapies like radiation were used only in a few cases, mainly when surgery could not completely remove the tumor or when aggressive features were present. Understanding the behavior of OFMTs is important to avoid overtreatment while ensuring proper care. Future research should focus on molecular profiling to help predict the behavior of these rare tumors and guide personalized treatment and follow-up strategies.

## 1. Introduction

Ossifying fibromyxoid tumors (OFMTs) are rare mesenchymal neoplasms first described by Enzinger et al. in 1989 [1]. These tumors exhibit intermediate biological behavior, ranging from benign to malignant forms [2]. While OFMTs are commonly found in the extremities and trunk, approximately 9–13% of cases occur in the head and neck region, including the oral cavity, scalp, calvarium, and skull base [3,4]. Clinically, OFMTs typically present as slow-growing, well-demarcated, painless masses, often rendering them challenging to distinguish from other benign lesions [5]. Histopathologically, OFMTs are encapsulated tumors composed of uniform polygonal to spindle-shaped cells within a fibromyxoid stroma. A distinctive rim of metaplastic lamellar bone is observed in approximately 70% of cases. Immunohistochemical analysis frequently shows positivity for markers such as the S-100 protein, vimentin, and occasionally desmin, indicative ofneuroectodermal or myoepithelial differentiation [6,7]. Recent genetic studies have revealed PHF1 rearrangements and occasional INI-1 deletions, providing molecular insights into the tumor’s pathogenesis [8]. Malignant variants are characterized by increased cellularity, mitotic activity, and nuclear atypia, correlating with a higher risk of recurrence and metastasis [9]. The diagnosis and management of OFMTs in the head and neck region are particularly challenging due to the anatomical complexity and functional significance of these areas. Given their rarity, OFMTs are frequently misdiagnosed as other neoplastic or reactive lesions [10,11]. Imaging modalities such as computed tomography (CT) or magnetic resonance imaging (MRI) may reveal nodular masses with peripheral ossification, aiding in diagnosis but lacking specificity [12]. Histological evaluation remains the diagnostic gold standard. However, differentiation from other entities, such as myoepithelial tumors, extra-skeletal myxoid chondrosarcomas, and schwannomas, requires careful consideration, particularly for atypical and malignant variants [13,14]. Surgical excision with negative margins is the cornerstone of treatment for OFMTs [15]. While most benign cases are effectively managed with surgery alone, atypical and malignant variants necessitate closer surveillance due to their increased recurrence potential and, in rare cases, distant metastasis [16]. Adjuvant therapies, including radiotherapy or chemotherapy, have a limited role but may be considered in cases of unresectable or metastatic disease [17]. Understanding the clinical and pathological spectrum of OFMTs is critical for accurate diagnosis and effective management. This systematic review synthesizes data on OFMTs in the head and neck region to enhance diagnostic accuracy by identifying key clinical and histopathological features, evaluating the efficacy of surgical and adjuvant treatments, and developing evidence-based recommendations for managing typical, atypical, and malignant forms. By addressing the gaps in current knowledge, this review aims to guide clinicians in diagnosing and treating these rare and challenging tumors.

## 2. Materials and Methods

This systematic review was conducted to comprehensively evaluate the clinical presentation, histopathological features, surgical management, and adjuvant therapies for ossifying fibromyxoid tumors (OFMTs) of soft parts in the head and neck, including specific regions such as the oral cavity, scalp, and calvarium. The methodology followed the Preferred Reporting Items for Systematic Reviews and Meta-Analyses (PRISMA) guidelines to ensure a rigorous and reproducible process, encompassing an exhaustive search strategy, structured inclusion and exclusion criteria, detailed data extraction, and a comprehensive synthesis of findings.

To identify all the relevant studies, searches were performed across four major electronic databases: PubMed/MEDLINE, Embase, Scopus, and Web of Science. These databases were selected for their extensive coverage of biomedical, clinical, and multidisciplinary research. The search strategy combined Medical Subject Headings (MeSH) terms and free-text keywords to maximize sensitivity and ensure the comprehensive coverage of the literature, addressing OFMTs in the head and neck region. The core search terms included “ossifying fibromyxoid tumor”, “head and neck neoplasms”, “oral neoplasms”, “scalp tumors”, “calvarial tumors”, and “skull base tumors”. Boolean operators (AND/OR) were employed to refine the search, and synonyms were incorporated to capture all possible variations in terminology. Filters were applied to select studies published between January 1989 and December 2024, encompassing both historical perspectives and the most up-to-date findings.

Screening and selection followed a rigorous multistage process. First, all retrieved records were reviewed by title and abstract to exclude irrelevant studies. The remaining full-text articles were assessed for eligibility based on predefined criteria. Two independent reviewers (G.S. and Z.S.) conducted the screening and selection independently. A third reviewer (S.M.) was consulted in cases of disagreement, ensuring the unbiased resolution of conflicts. The inclusion criteria specified that studies must report OFMTs localized to the oral cavity, scalp, calvarium, or other head and neck regions, provide detailed clinical and histopathological data (including cytologic atypia, ossification patterns, and mitotic activity), describe treatment modalities (surgical intervention and adjuvant therapies), and include outcome measures such as recurrence rates and follow-up. Eligible study designs included case reports, case series, retrospective cohort studies, and prospective observational studies. Articles not meeting these criteria, including reviews, editorials, non-English studies without translation, and reports on OFMTs in regions outside of the head and neck, were excluded.

A manual search was also conducted by cross-referencing the bibliographies of all the included studies and related review articles. This step was essential to identify additional relevant studies that may not have been indexed in the primary database searches. All studies identified through cross-referencing underwent the same rigorous evaluation and selection process to ensure consistency.

Data extraction was performed systematically using a standardized and piloted form to ensure uniformity and reduce errors. Key information extracted from each study included patient demographics (age and sex), tumor characteristics (location, size, histopathological subtype, cytological features, ossification patterns), treatment details (surgical margins, techniques, and adjuvant therapies such as radiotherapy or chemotherapy), and clinical outcomes (recurrence rates and follow-up). Extracted data were independently reviewed and verified by G.S. and Z.S., and discrepancies were resolved through discussion or consultation with S.M. The extracted information was collated into a tabular format to facilitate comparison and synthesis (Table 1).

The included studies were synthesized into a comprehensive narrative and tabular summary (Table 2), focusing on key findings such as the influence of tumor location and histopathological features on clinical outcomes, the role of margin status in recurrence, and the effectiveness of adjuvant therapies.

**Figure 1:**
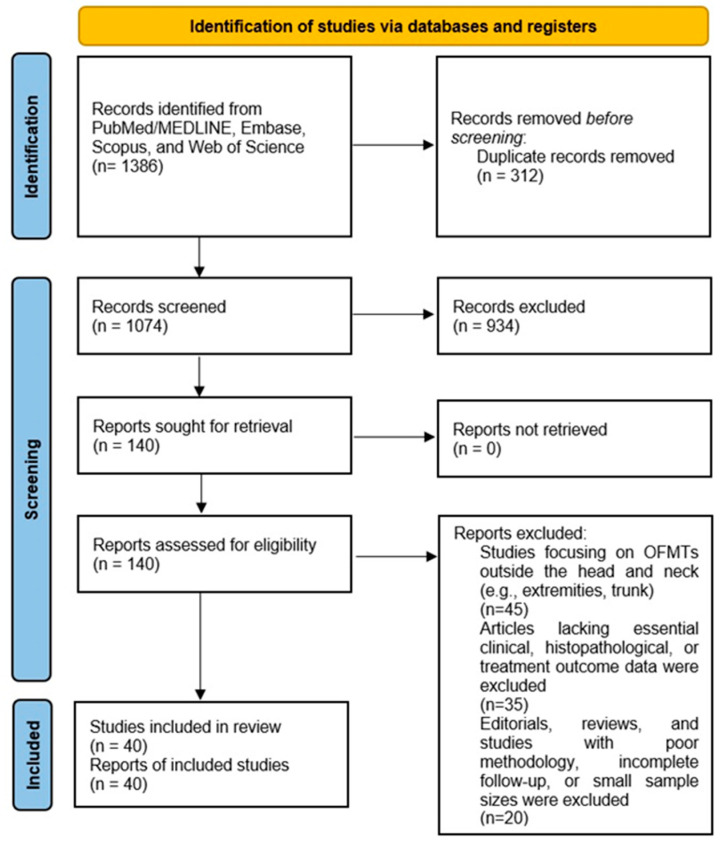
The PRISMA flow diagram illustrates the systematic review process for ossifying fibromyxoid tumors (OFMTs) in the head and neck. The diagram outlines the stages of study selection, including the number of records identified through database searches, duplicates removed, records screened, full-text articles assessed for eligibility, and the final number of studies included. Reasons for exclusion at each stage are also documented, ensuring transparency and reproducibility of the review process.

To visualize the selection process, a PRISMA-compliant 2020 flow diagram was created, illustrating the number of records identified, screened, excluded, and included at each stage of the review, along with reasons for exclusion during full-text review. This ensured the transparency and reproducibility of the study selection process (Figure 1).

This systematic review was conducted following the PRISMA 2020 checklist (Supplementary File S1) to ensure methodological rigor and transparency [42]. Predefined inclusion and exclusion criteria were applied, and study selection was performed in accordance with the PRISMA flow diagram recommendations.

This systematic review provides the most comprehensive evaluation to date of OFMTs in the head and neck region, incorporating data from multiple databases and manual searches to inform clinical management strategies. The findings underscore the importance of meticulous surgical planning, the potential utility of adjuvant therapies in specific cases, and the need for further research into the long-term outcomes and prognostic factors associated with this rare tumor entity.

## 3. Risk of Bias Assessment

The risk of bias for the included studies was evaluated using the Newcastle–Ottawa Scale (NOS), a validated tool for assessing the methodological quality of observational studies. The NOS assigns a maximum of 9 points across three key domains: selection (4 points), comparability (2 points), and Eexposure/outcome (3 points). Two independent reviewers performed the assessment, with a third reviewer resolving any discrepancies. Studies scoring ≥ 7 were classified as a low risk of bias, those with 4–6 points as moderate risk, and those scoring < 4 as high risk of bias.

## 4. Results

The systematic search yielded an initial dataset of 1386 articles across all databases. After removing duplicates (*n* = 312), 1074 unique records underwent title and abstract screening. Of these, 145 articles were selected for full-text review. Following the application of inclusion and exclusion criteria, a total of 40 studies were deemed eligible for inclusion in the systematic review. The dataset analyzed a total of 99 patients across 40 articles published between 1989 and 2024, representing a wide range of cases involving various head and neck regions, such as the oral cavity, scalp, and calvarium.

The ages of the patients ranged from a 3-week-old infant to 88 years, with a median age of 47 years, a mean of 46.75 years (±12.23), and an interquartile range of 39 to 52 years. Among the documented cases, males represented 63 cases (63.64%), while females accounted for 36 cases (36.36%), revealing a modest male predominance.

The anatomical locations of the lesions were diverse. The neck was the most reported site, documented in eighteen cases (6.06%), followed by other frequent locations such as the cheek, lip, and nasal cavity. Notably, the scalp was documented in six cases (6.06%), and calvarial locations in two cases (2.02%). Scalp lesions were associated with subcutaneous masses and were managed predominantly with local excision, demonstrating favorable clinical outcomes without recurrence. The single calvarial case involved a cystic scalp mass requiring a craniectomy and methylmethacrylate cranioplasty, with no recurrence during follow-up, reflecting the efficacy of combined surgical and reconstructive approaches for such cases.

In terms of clinical presentation, most cases (14 cases, 14.14%) were characterized as “slow-growing masses”, which appeared as the predominant feature across the dataset. This aligns with the benign nature of most lesions documented in the studies. Other clinical presentations include painless swelling, subcutaneous masses, exophytic lesions, congestion, and indurated masses.

Regarding surgical treatments, an “excisional biopsy” (local excision) was the most frequently employed surgical treatment, documented in 28 cases (28.28%), indicating its widespread acceptance and effectiveness. Mitotic activity was assessed in 50 cases (50.51%), with 23 cases (23.23%) reporting “none detected” and the remainder demonstrating low mitotic indices (≤2 mitoses per 10 High-Power Fields—HPFs), further corroborating the benign nature of most lesions. Similarly, atypia was absent in 31 cases (31.31%), reinforcing the non-aggressive characteristics of these conditions.

Recurrence rates were low, with 31 cases (31.31%) confirming no recurrence following treatment. Of the nine cases (9.09%) reporting recurrence, localized recurrence was documented in two cases (2.02%). In approximately 59.60% of cases, information regarding recurrence was not explicitly reported in the original studies. Systemic spread was exceedingly rare, with no metastasis reported in 40 cases (40.40%). Follow-up durations were inconsistently documented, with 13 cases (13.13%) providing no follow-up data. Among those that did, follow-up periods ranged from 6 months to 33 years, with a mean follow-up of 6.21 years (±3.50), highlighting variations in post-treatment monitoring practices.

Adjuvant therapies were rarely utilized, with 84 cases (84.85%) indicating that no additional treatments were administered. When adjuvant therapies were applied (15 cases, 15.15%), they were limited to select cases and included radiation therapy or chemotherapy, often for lesions with atypical features or recurrences. This suggests that most cases were effectively managed through surgical intervention alone, reflecting the non-invasive nature of most of these lesions.

## 5. Risk of Bias Results

The NOS assessment revealed variability in methodological quality among the 40 included studies, with a mean score of 5.22 and a median score of 6.0.

- Low risk of bias (≥7 points): four studies.
- Moderate risk of bias (4–6 points): thirty-one studies.
- High risk of bias (<4 points): five studies.

Most studies demonstrated a moderate risk of bias, primarily due to limitations in follow-up duration, case definition, and the standardization of treatment reporting. However, a subset of high-quality studies with detailed methodologies and long-term follow-up significantly strengthened the overall reliability of the findings. Despite some methodological limitations, the data collected provided valuable insights into the diagnosis and management of ossifying fibromyxoid tumors (OFMTs) in the head and neck region.

### Illustrative Case

A 15-year-old male presented with a progressively enlarging mass in the right parietal region of the skull. The lesion had been gradually increasing in size over several years, raising concerns about its nature. The patient reported no associated symptoms such as pain, neurological deficits, or systemic manifestations. His medical history was unremarkable, with no history of head trauma, prior surgeries, or chronic illnesses.

Physical examination revealed a firm, non-tender swelling closely adherent to the underlying bone. No erythema, warmth, or skin abnormalities were noted over the lesion. Neurological examinations showed no deficits, with normal cranial nerve function, symmetrical motor strength, and preserved sensory responses. Based on clinical findings, imaging studies were requested for further evaluation.

**Figure 2:**
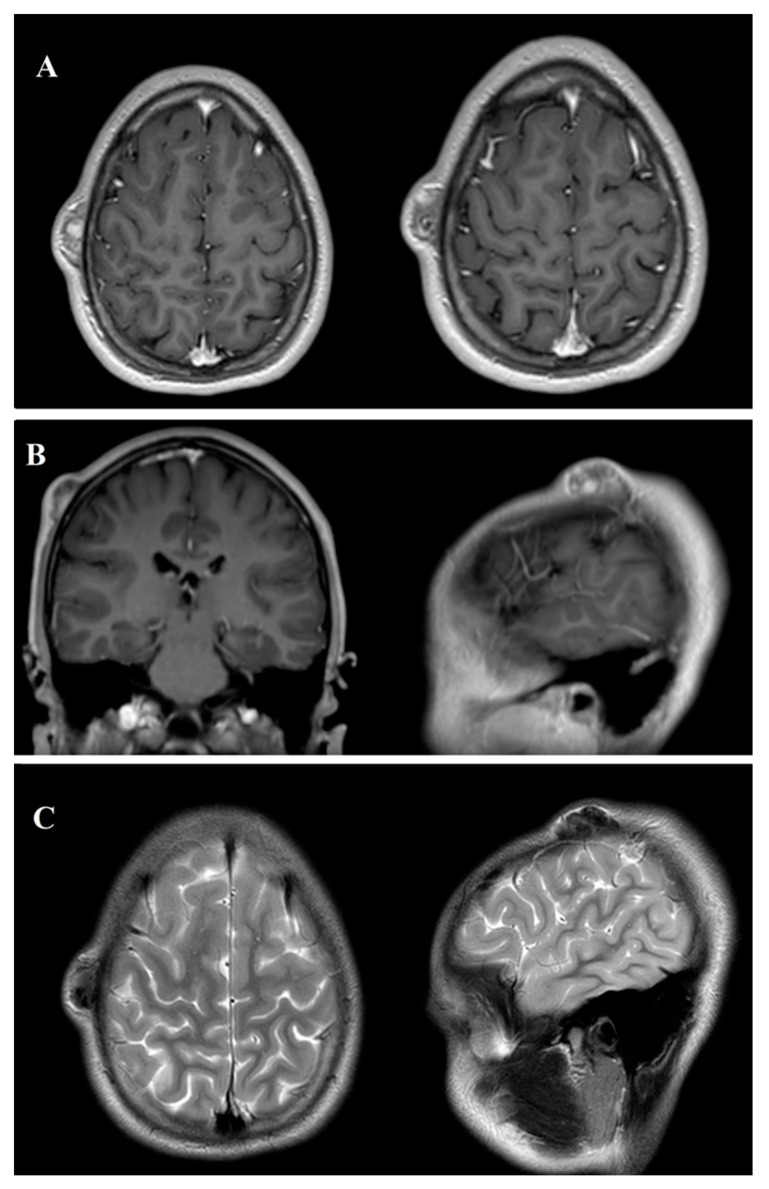
Preoperative magnetic resonance imaging (MRI) of an ossifying fibromyxoid tumor (OFMT) in the head and neck region, displayed in three key panels. Panel (**A**) shows axial T1-weighted sequences with gadolinium (Gd) contrast, highlighting the well-defined, expansile nature of the tumor with marked contrast enhancement, indicative of vascularity or metabolic activity. Panel (**B**) presents coronal and sagittal T1-weighted sequences with Gd contrast, offering a multi-planar view of the lesion’s relationship with the surrounding tissues and its anatomical boundaries. Panel (**C**) illustrates T2-weighted axial and sagittal sequences, where the tumor appears as a hyperintense mass, providing additional details on internal composition and fluid content. The combined imaging approach enables the precise characterization of the lesion’s location, structure, and extent, supporting preoperative planning and surgical decision-making.

A preoperative MRI of the brain and cranial structures was performed using multiple sequences (SE, TSE, DWI, FLAIR, STIR, FFE, and 3D-TFE) before and after intravenous contrast administration. The imaging revealed a well-defined, expansile lesion measuring 30 mm anteroposteriorly and 9 mm craniocaudally, located in the subcutaneous soft tissues and closely adherent to the outer table of the right parietal bone. The lesion displayed marked contrast enhancement, suggestive of vascularity or active metabolic processes, with evidence of internal calcifications or hemosiderin deposits, indicating chronicity or previous microhemorrhages. Importantly, there was no invasion of the cranial bone or extension into adjacent soft tissues. The brain parenchyma, ventricular system, and major intracranial structures appeared normal, with no significant signal abnormalities or mass effects (Figure 2).

**Figure 3:**
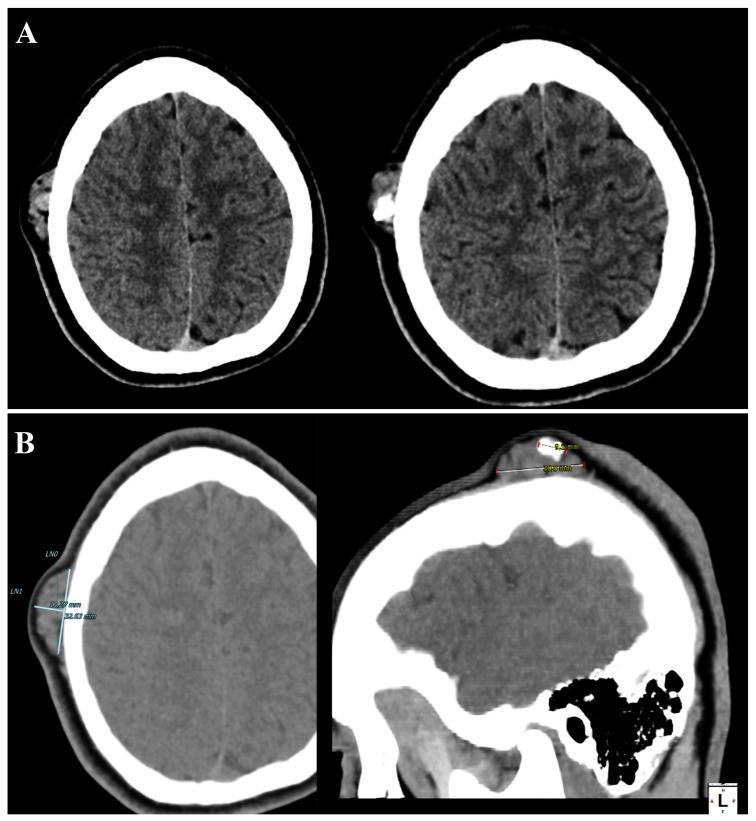
Computed tomography (CT) imaging of an ossifying fibromyxoid tumor (OFMT) in the head and neck region, presented through two key panels. Panel (**A**) shows axial CT slices, highlighting the lesion as a soft-tissue density mass with a well-defined outline and a central calcified component. Linear measurements between reference points LN0 and LN1 indicate transverse dimensions of approximately 32.63 mm and 11.27 mm, respectively, likely representing the lesion’s full width and a perpendicular component. Panel (**B**) displays sagittal reconstructions using a bone window algorithm, showing the intralesional calcified component with a height of 9.5 mm and a length of 39.8 mm. These measurements provide critical spatial characterization of the lesion, supporting surgical planning and preoperative risk assessment. The patient underwent surgical excision of the lesion under local anesthesia with sedation. A linear incision was made over the parietal region, and the lesion was carefully dissected from the surrounding soft tissues and the external cortical surface of the parietal bone. Intraoperatively, the mass was noted to be bilobed, firm, and cystic, measuring 1.5 cm at its largest diameter. There was no involvement of the underlying cranial bone, consistent with preoperative imaging findings. The lesion was completely excised and sent for histopathological examination.

A high-resolution computed tomography (CT) scan of the head confirmed the MRI findings. The lesion, located in the extracranial soft tissues of the right parietal region, measured 30 mm in diameter with a maximum thickness of 14 mm. It exhibited a soft-tissue density with a central calcified area measuring approximately 10 mm and maintained a broad base of attachment to the external surface of the parietal bone, without evidence of cortical disruption or infiltration into the cranial bone (Figure 3). These findings supported the diagnosis of a benign-appearing extracranial lesion and informed the decision for surgical excision.

The histological examination confirmed the lesion as an ossifying fibromyxoid tumor (OFMT), a rare mesenchymal neoplasm with low malignant potential. The tumor displayed hallmark features consistent with its classification, including low cellularity within a fibromyxoid stroma and the absence of nuclear atypia. Mitotic activity was notably low, with fewer than one mitosis per fifty High-Power Fields (HPFs), supporting its indolent nature.

Morphologically, the tumor was composed of lobules of uniform, round to spindle-shaped neoplastic cells with bland, round to ovoid nuclei, and scant, pale eosinophilic cytoplasm, as observed at 20× magnification. A multinodular growth pattern was present, often accompanied by areas of metaplastic bone formation, further characteristic of ossifying fibromyxoid tumors.

Immunohistochemical analysis provided critical diagnostic insights. The tumor cells showed strong positivity for SOX10 (20× magnification) and S100, confirming neural crest origin. Both nuclear and cytoplasmic immunopositivity for S100 were evident, which is a typical feature of this tumor type. Conversely, the tumor was negative for panCK, CD68, and actin, excluding epithelial, macrophage, and smooth muscle components, respectively. The absence of staining for alpha-smooth muscle actin (SMA) at 20× magnification reinforced the exclusion of smooth muscle differentiation. Similarly, negative staining for CD68 (20× magnification) ruled out the presence of macrophage or histiocytic elements.

**Figure 4:**
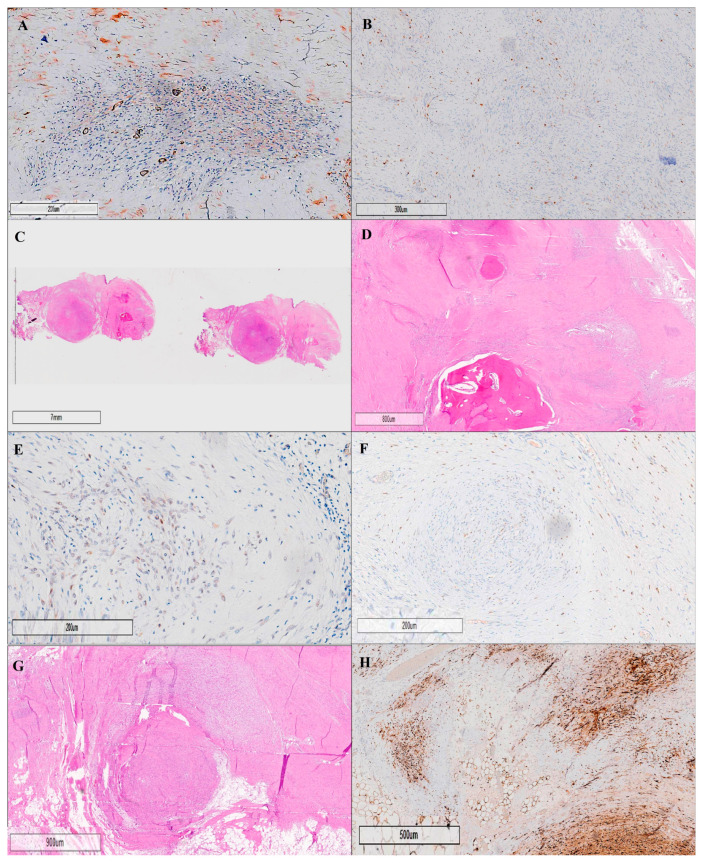
Histopathological and immunohistochemical analyses of an ossifying fibromyxoid tumor (OFMT) are illustrated through eight key panels, each labeled (**A**–**H**), highlighting essential diagnostic features. Panel (**A**) shows negative staining for alpha-smooth muscle actin (SMA) at 20× magnification, excluding the presence of smooth muscle differentiation, while panel (**B**) displays negative staining for CD68 at 20× magnification, ruling out macrophage or histiocytic components. The general architecture of the tumor is presented in panel (**C**), which provides a low-power (10×) hematoxylin and eosin (H&E) overview of the tumor’s structure. A higher magnification (20×) view is seen in panel (**D**), revealing a multinodular growth pattern of neoplastic cells within a fibromyxoid stroma, along with characteristic areas of metaplastic bone formation. Panel (**E**) highlights the immunohistochemical staining for SMARCB1 (INI1) at 20× magnification, demonstrating a mosaic pattern of expression, a distinctive molecular feature of OFMTs. Panel (**F**) demonstrates weak-to-moderate nuclear positivity for SOX10 at 20× magnification. The detailed cellular composition is further exemplified in panel (**G**), which illustrates lobules of uniform, round to spindle-shaped neoplastic cells with bland, round to ovoid nuclei, and scant, pale eosinophilic cytoplasm (H&E, 20×). Finally, panel (**H**) reveals nuclear and cytoplasmic immunopositivity for S-100 at 20× magnification, a hallmark diagnostic feature of OFMTs. Collectively, these panels provide a comprehensive visual overview of the key histopathological and immunohistochemical features of ossifying fibromyxoid tumors, supporting accurate diagnosis and differentiation from other soft-tissue neoplasms.

Another crucial immunohistochemical finding was the expression of SMARCB1 (INI1) in a mosaic pattern (20× magnification), which is a known characteristic in certain mesenchymal neoplasms and provides an additional diagnostic clue (Figure 4).

Importantly, no residual tumor was observed at the surgical margins, indicating a complete excision. This is a significant prognostic factor given the tumor’s low malignant potential. Taken together, the morphological, immunohistochemical, and mitotic activity findings provide a comprehensive diagnostic confirmation of an ossifying fibromyxoid tumor, supporting its designation as a low-grade neoplasm with a favorable clinical outcome.

This case underscores the importance of multimodal imaging in the management of rare cranial neoplasms. The patient’s complete recovery and favorable prognosis reflect the efficacy of a multidisciplinary approach involving neurosurgery, radiology, and pathology.

Regular follow-up will monitor for recurrence, although the tumor’s benign characteristics and complete surgical resection with clear margins suggest a low risk of regrowth. This case contributes to the limited literature on ossifying the fibromyxoid tumors of soft parts and demonstrates the value of advanced imaging in guiding diagnosis and treatment.

## 6. Discussion

OFMTs are a rare and morphologically heterogeneous group of mesenchymal neoplasms with biological behavior that ranges from benign to malignant [18]. First described by Enzinger et al. in 1989 [1], these tumors have drawn significant interest due to their unpredictable clinical course, histopathological overlap with other neoplasms, and their relatively recent molecular characterization [43]. The rarity of OFMTs, particularly in the head and neck region, which accounts for only 9–13% of all reported cases, presents unique diagnostic and management challenges [19,27,31]. This systematic review of 40 articles analyzing a total of 99 patients provides an in-depth analysis of the clinical, histopathological, molecular, and therapeutic characteristics of these tumors, offering new perspectives for clinical practice and future research.

## 7. Histopathological and Molecular Insights

Histopathologically, OFMTs exhibit a distinctive lobulated and encapsulated architecture, often surrounded by a fibromyxoid stroma containing uniform polygonal to spindle-shaped cells [44]. A defining feature of these tumors is the presence of a rim of metaplastic lamellar bone, seen in 70% of cases, which helps differentiate them from other sarcomas and soft-tissue neoplasms [25,28].

The immunohistochemical profile of OFMTs is typically positive for S-100 and vimentin, and this positivity may help with the diagnosis [6]. The absence of S-100 positivity may be associated with a more aggressive disease [18]. Indeed, although clear evidence supporting this assertion is still lacking, it appears that when the tumor exhibits malignant behavior, S-100 expression is often focal or absent, rather than diffused as seen in benign subtypes [18]. Additionally, in such cases, the presence of epithelial markers, such as cytokeratins, is more frequently observed [8]

Benign, atypical, and malignant subtypes are differentiated by key histological features, including cellularity, nuclear atypia, mitotic activity, and the extent of infiltrative growth [18]. The threshold for malignancy is typically set at more than 12 mitoses per 50 High-Power Fields (HPFs) [27,34,38]. The results of this systematic review revealed that mitotic activity was reported in 50% of the studies, with 23.23% of cases showing no detectable mitotic figures and the remainder demonstrating low mitotic indices (≤2 mitoses per 10 HPFs), further supporting the benign nature of most lesions. Atypia was reported to be absent in 31.31% of cases, reinforcing the non-aggressive behavior of most OFMTs.

The origin of these tumors remains uncertain. Although a Schwannian origin has been hypothesized, partly due to the positivity for S-100, an interesting aspect is the potential expression of CD56 and CD99 in some tumors [25]. These markers are often associated with the diagnosis of tumors of neuroectodermal origin, suggesting that their expression could be linked to a neuronal cell origin. However, the real origin of these tumors remains unknown.

At the molecular level, genetic alterations have emerged as critical diagnostic and prognostic markers. PHF1 rearrangements and INI-1 deletions have been identified in several studies, with PHF1 rearrangements observed in up to 50% of OFMT cases. These molecular abnormalities are more common in atypical and malignant variants, suggesting their role in tumor progression [30]. Genetic profiling, as reported by Graham et al. and Antonescu et al. [8,45], reinforces the view that OFMTs are a genetically distinct entity. These molecular insights are crucial for differentiating OFMTs from other soft-tissue sarcomas and may provide a basis for future targeted therapeutic interventions.

## 8. Clinical and Radiological Characteristics

Clinically, OFMTs in the head and neck region present as slow-growing, painless masses [22]. The results of this systematic review revealed that 14.14% of cases were described as “slow-growing masses”, making them the most frequently reported clinical presentation. This aligns with the indolent course observed in most OFMTs. While most cases are asymptomatic, tumors located in anatomically complex regions such as the neck, nasal cavity, scalp, and calvarium may present unique challenges [24,30,39]. Scalp lesions, as in our case, were reported in 3.03% of cases, while calvarial lesions were noted in 1.01% of cases. Scalp lesions, often managed with local excision, had no recorded cases of recurrence or metastasis, indicating a positive prognosis following surgical intervention. Calvarial lesions, which pose a higher surgical challenge due to the involvement of bony structures, often require more complex procedures. For instance, the documented calvarial case required a craniectomy and reconstruction using methylmethacrylate cranioplasty, with no recurrence noted during the follow-up period.

Radiologically, computed tomography (CT) and magnetic resonance imaging (MRI) are key tools in the diagnostic process. CT imaging often reveals a thin rim of peripheral ossification, while MRI typically shows a well-circumscribed lesion with isointense-to-hyperintense signals on T1- and T2-weighted images [17,21]. Lytic bone destruction, extracranial extension, or intracranial extension are features that may be present in more aggressive lesions, as seen in malignant subtypes or calvarial involvement [20,23]. This finding underscores the importance of a multidisciplinary approach involving radiologists, pathologists, and surgeons to ensure accurate diagnosis and treatment planning.

## 9. Therapeutic Approaches

Surgical excision remains the gold standard for all OFMT subtypes [29,33]. The systematic review revealed that local excision (referred to as excisional biopsy in some studies) was the most performed procedure, utilized in 28.28% of cases. The rationale for complete surgical excision lies in its ability to achieve clear margins, thereby minimizing the risk of recurrence. In benign subtypes, complete excision is often curative, with no further treatment required [32,35,46]. For atypical and malignant variants, more aggressive surgical strategies are warranted. En bloc resection is recommended for malignant subtypes, particularly those with infiltrative margins or evidence of bony destruction [10,13,15,38]. Incomplete resection has been associated with higher rates of recurrence and poorer outcomes [27,29]. Recurrence rates across the histological spectrum were reported in 9.09% of cases, with 2.02% of cases showing localized recurrence. Importantly, 40.40% of cases had no recorded metastasis. The low rate of systemic spread highlights the predominantly indolent nature of OFMTs, with only 5% of malignant subtypes exhibiting metastatic potential.

## 10. Role of Adjuvant Therapy

While surgery remains the primary treatment for OFMTs, the role of adjuvant therapies, particularly radiotherapy and chemotherapy, is evolving. The systematic review revealed that 15.15% of cases involved the use of adjuvant therapies, which included radiotherapy and chemotherapy. These therapies are not routinely employed for benign subtypes but are considered in cases where complete surgical excision is not feasible or when dealing with malignant variants [37,47,48]. Radiotherapy has shown promise in controlling local disease in cases where surgical margins are close or positive [40]. It has also been used as a salvage therapy in recurrent or inoperable lesions [35]. However, the overall evidence supporting its efficacy remains limited, and further studies are needed to establish its role in clinical practice.

The application of adjuvant chemotherapy remains controversial. Its use is generally reserved for metastatic disease or for lesions with high mitotic activity and other aggressive features [49]. Given the rarity of malignant OFMTs, data on chemotherapy’s efficacy remain scarce, and its role is considered supplementary rather than primary.

## 11. Prognostic Factors and Follow-Up

The prognosis of OFMTs depends on the histological subtype, molecular profile, and the adequacy of surgical resection [26]. Typical OFMTs have an excellent prognosis, with recurrence occurring in only 9.09% of cases [3,29]. Malignant subtypes, however, have a higher risk of recurrence and metastasis [27,31,41]. The presence of PHF1 rearrangements and INI-1 deletions may further stratify patients into high- or low-risk categories, providing a molecular basis for risk assessment [8,30]. These findings suggest that molecular diagnostics should be integrated into follow-up protocols for patients with atypical or malignant subtypes.

Follow-up protocols should be stratified based on histological and molecular risk factors [36]. The systematic review revealed follow-up durations ranging from 6 months to 33 years, with a mean of 6.21 years (±3.50). Imaging modalities such as CT or MRI should be employed periodically to detect local recurrence or distant metastasis, particularly for high-risk subtypes. Surveillance should be more frequent in patients with atypical or malignant OFMTs, especially when molecular markers indicate a higher risk of recurrence.

## 12. Challenges, Limitations, and Future Directions

Despite advances in the characterization and management of OFMTs, significant challenges remain. The rarity of these tumors, coupled with their histopathological overlap with other soft-tissue neoplasms, underscores the need for heightened clinical awareness and expertise in diagnostic pathology. Standardizing the role of adjuvant therapies, particularly radiotherapy, remains an area of ongoing investigation. Further multicenter studies with larger patient cohorts are essential to clarify the role of molecular diagnostics, surgical margins, and adjuvant therapies in patient outcomes. Although NOS provides a validated framework for assessing the quality of observational studies, it is important to recognize its limitations, including the subjective interpretation of certain criteria and the lack of a universally accepted standardized scoring system. Future research should prioritize the development of molecular-targeted therapies, particularly for malignant subtypes where recurrence and metastasis pose significant risks. Additionally, long-term follow-up studies are essential to better understand the natural history of OFMTs, recurrence patterns, and survival outcomes. The use of molecular biomarkers, such as PHF1 and INI-1, in stratifying patients and personalizing follow-up protocols may lead to improved patient care.

## 13. Conclusions

OFMTs in the head and neck region remain a diagnostic and therapeutic challenge due to their rarity and variable biological behavior. This systematic review highlights key clinical, radiological, molecular, and therapeutic findings. The role of surgery as the primary treatment remains undisputed, while the use of adjuvant therapies, particularly radiotherapy, requires further validation. The findings underscore the importance of a multidisciplinary approach, with molecular profiling emerging as a crucial tool for personalized patient management.

## Figures and Tables

**Table 1:** Summary of individual patient data for ossifying fibromyxoid tumors (OFMTs) in the head and neck region. The table includes details on patient demographics, tumor location, clinical presentation, surgical treatment, histopathological findings (mitotic activity and atypia), recurrence, metastasis, follow-up duration, and use of adjuvant therapies. This comprehensive dataset provides insight into the clinical and pathological spectrum of OFMTs, aiding in diagnosis and management strategies.

Authors and Year	Number of Patients	Age (Years)	Sex	Location	Clinical Presentation	Surgical Treatment	Mitotic Activity	Atypia	Recurrence	Metastasis	Follow-Up (Years)	Adjuvant Therapy
Enzinger FM et al. (1989) [1]	8	14–79 (mean 47)	64% M	Head and neck, various sites	Slow-growing painless mass	Local excision (majority of cases)	N/D	N/D	N/D	No	1–32 (mean 6.2)	N/D
Schofield JB et al. (1993) [6]	4	41	M	Inner cheek	Slow-growing mass	N/D	−	−	No	No	1–10 (median 7)	N/D
		78	M	Neck	Slow-growing mass	N/D	N/D	N/D	No	No		N/D
		39	M	Lip	Slow-growing mass	N/D	0–11/10 High-Power Fields (HPFs)	−	No	No		N/D
		49	M	Pretracheal	Slow-growing mass	N/D	N/D	N/D	No	No		N/D
Williams SB et al. (1993) [7]	9	51	F	Posterior neck	Slow-growing mass	Local excision	−	−	No	No	2	N/D
		52	M	Submental area	Slow-growing mass	Local excision	−	−	No	No	N/D	N/D
		39	M	Chin	Slow-growing mass	Local excision	−	−	No	No	2	N/D
		67	F	L mandible vestibule	Slow-growing mass	Local excision	−	−	No	No	1.5	N/D
		29	M	L lateral max and nasal bone	Slow-growing mass	Local excision	−	−	No	No	1.5	N/D
		37	M	Soft palate	Slow-growing mass	Local excision	−	−	No	No	3	N/D
		66	M	Scalp	Slow-growing mass	Local excision	−	−	No	No	2	N/D
		75	M	L neck	Slow-growing mass	Local excision	−	−	No	No	1	N/D
		58	F	L parapharynx	Slow-growing mass	Local excision	+	+	Yes (2)	No	2.1	N/D
Williams RW et al. (1994) [10]	1	35	M	Parotid/zygomatic arch region	Slow-growing mass	Wide local excision	+	+	Yes (3)	No	24	N/D
Ng WK et al. (1995) [11]	1	52	M	R nostril, middle meatus	Swelling, intermittent epiphora	N/D	N/D	N/D	N/D	N/D	N/D	N/D
Thompson J et al. (1995) [12]	1	35	M	L nasal cavity	Congestion and pain	Partial removal	–	–	Yes	No	N/D	Radiation therapy
Lax (1995) [13]	1	50	M	R thyroid lobe	Nodular enlargement	Total thyroidectomy	+	–	No	No	3	External irradiation of neck
Zamecnik M et al. (1997) [14]	2	71	M	Neck	Subcutaneous mass	Surgical removal	+	+	Yes (2)	No	4	N/D
		45	F	Neck	Subcutaneous mass	Surgical removal	+	+	Yes	Yes (lung)	DOD	Chemotherapy
Ekfors TO et al. (1998) [4]	2	63	M	Neck	Subcutaneous mass	N/D	+	–	No	No	N/D	N/D
		2	F	Head	Subcutaneous mass	N/D	–	–	No	No	N/D	N/D
Paschen C et al. (2001) [16]	1	12	M	Nasal cavity and paranasal sinus	Nasal congestion	Local excision	–	–	No	No	N/D	N/D
Folpe AL et al. (2003) [18]	9	14–80 (median 49)	56% M	Head and neck, various sites	Subcutaneous mass	Wide excision (majority of cases)	1 patient	–	Yes (2 in single patient)	1 patient (leg)	5–20 (mean 4.7)	N/D
Al-Mazrou KA (2004) [19]	1	3 wk. infant	M	L nasomaxillary fold	Slight fullness	Local excision	–	–	No	No	0.5	N/D
Mollaoglu N et al. (2006) [20]	1	13	M	L side of mandible	Rapid swelling	Local excision	–	–	No	No	N/D	N/D
Park DJ et al. (2006) [21]	1	81	F	R orbit	Diplopia, pain, swelling	Local excision	–	–	Yes (2)	No	6	Radiation therapy
Seykora JT et al. (2006) [22]	1	67	F	Scalp	Multilobular and cystic mass	Wide local excision	2/10 HPFs	–	No	No	8	N/D
Suehiro K et al. (2006) [23]	1	38	F	Scalp	Subcutaneous mass	Local excision	5/10 HPFs	–	Yes (3)	Yes (lung and brain)	DOD	N/D
Blum A et al. (2006) [24]	1	49	F	Nasal septum	Nasal congestion and swelling	Local excision	–	–	No	No	0.08	N/D
Miliaras D et al. (2007) [25]	1	39	M	Mandibular symphysis skin	Slow-growing subcutaneous mass	Local excision	–	–	No	No	1	N/D
Hirose T et al. (2007) [26]	2	42	M	Nasal vestibule	N/D	Local excision	+	+	Yes (2)	No	17	N/D
		54	M	Left supraclavicular region	Small nodule	Local excision	–	–	No	No	0.5	N/D
Sharif MA et al. A et al. (2008) [9]	1	14	F	Between buccal and gingival mucosa	Slowly growing gingival mass	Local excision	–	–	No	No	N/D	N/D
Miettinen M et al. (2008) [27]	20	21–81 (median 50)	62% M	9 neck, 3 scalp, 1 lower lip, 7 various sites	Variable	Local excision	0–41/50 HPFs	–	22% (≥1)	No	2–61 (mean 13)	N/D
Nonaka CF et al. (2009) [28]	1	21	F	R posterior mandibular gingiva	Painless exophytic mass	Local excision	–	–	No	No	0.6	N/D
Graham RP et al. (2011) [8]	9	39–63 (median 52)	52% M	Head and neck, various sites	N/D	N/D	N/D	N/D	N/D	N/D	1–2.4	N/D
Kondylidou-Sidira et al. (2011) [29]	1	24	M	Zygomatomaxillary buttress	Subcutaneous lump	Local excision	–	–	No	No	2	N/D
Gebre-Mehdin et al. (2012) [30]	4	47	F	Temple	N/D	N/D	–	–	No	No	N/D	N/D
		59	M	Neck	N/D	N/D	+	Yes (1)	Yes (rib)	N/D		N/D
		42	M	Neck	N/D	N/D	+	No	No	N/D		N/D
		43	M	Paralaryngeal	N/D	N/D	+	No	No	N/D		N/D
Ohta et al. (2013) [31]	1	26	M	Tongue	Painless mass	Local excision	>2/10 HPFs	–	Yes (1)	No	4	N/D
Ottoman B (2015) [32]	1	72	M	L posterior maxilla	Exophytic mass	N/D	–	–	No	No	N/D	N/D
Titsinides, S. et al. (2017) [33]	1	13	Male	Left retromolar triangle	Painless, hard, and non-moveable mass	Excisional biopsy	-	N/D	No	No	4 yr	N/D
Dantey, K. et al. (2017) [34]	1	33	Male	Parotid gland	N/D	Surgical resection	3/10 HPFs	+	No	No	33 mo	(Not needed) free of disease
Velasco, I.A. et al. (2018) [17]	1	52	Male	Right Submandibular region	Right neck swelling	Local excision	-	-	No	No	0.5	N/D
Kasai, K. et al. (2019) [35]	1	44	Female	Maxillary bone	Swelling	Local excision	N/D	N/D	No	No	N/D	N/D
Morita, Y. et al. (2021) [36]	1	88	Female	Maxilla gingiva	Painless swelling; pedunculated mass	Local excision	N/D	-	No	No	1 yr	N/D
Beyer, S. et al. (2021) [37]	1	29	Female	Right inferior frontal brain lobe	Enhancing mass with surrounding edema	Total neurosurgical resection	3/10 HPFs	+	No	N/D	5.5 yr	Fractionated SRT (FSRT)
Pérez-de-Oliveira, M.E. et al. (2021) [3]	1	45	Female	Anterior buccal mucosa	Painless nodule	Local excision	<2/10 HPFs	Mild pleomorphism	No	N/D	7 yr	N/D
Hachmann, J.T. and Graham, R.S. (2021) [38]	1	25	Female	Left parietal calvaria	Cystic scalp mass	Left parietal craniectomy and methylmethacrylate cranioplasty	40/50 HPFs	Moderate pleomorphism	No	No	Serial surveillance	N/D
Moriya, T. et al. (2021) [35]	1	63	Male	Right buccal mucosa	Hard swollen mass	Local excision	8/50 HPFs	+	No	No	1 yr	N/D
Alfozan, M. et al. (2023) [37]	1	41	Male	Right infratemporal fossa	Right preauricular swelling	Surgical removal	N/D	Minimal pleomorphism	Yes	Yes (cervical neck)	1 yr	Radiation therapy
Kinoshita, I. et al. (2023) [39]	1	54	Male	Right anterior nostril (nasal ala)	Right nasal swelling and mass	Endoscopic nasal surgery	-	-	No	N/D	N/D	N/D
San Millán-González, M. et al. (2024) [40]	1	54	Male	Right supraclavicular region	Well-defined indurated mass	Right cervicotomy	N/D	N/D	N/D	N/D	N/D	N/D
Nivedha G and Bhaskarashenoy, M. (2024) [41]	1	59	Female	Right mandibular region	Painless swelling	Curettage	<2/50 HPFs	+	N/D	N/D	Under follow-up	N/D

Abbreviations: N/D = Not Documented; HPFs = High-Power Fields; SRT = Stereotactic Radiotherapy; DOD = Dead of Disease; L = Left; R = Right; mo = Months; yr = Year(s).

**Table 2:** Aggregated analysis of ossifying fibromyxoid tumors (OFMTs) in the head and neck. This table summarizes key patient and tumor characteristics, including the total number of patients, age distribution, sex ratio, lesion location, clinical presentation, surgical treatment, mitotic activity, atypia, recurrence, metastasis, follow-up duration, and use of adjuvant therapies. The data highlight the variability in tumor behavior and treatment outcomes, offering guidance for clinical decision-making.

Variable	Description
Total Number of Patients	99
Age (years)	Range: 3 weeks–88 years; median: 47; mean: 46.75; SD: 12.23; IQR: 39–52.
Sex	Male: 63 (63.64%); female: 36 (36.36%).
Location of Lesions	Neck (18, 18.18%); scalp (6, 6.06%); calvarium/Skull (2, 2.02%); inner cheek (4, 4.04%); lip (2, 2.02%); pretracheal region (1, 1.01%); posterior neck (1, 1.01%); submental area (1, 1.01%); chin (1, 1.01%); mandibular vestibule (1, 1.01%); maxilla/nasal bone (1, 1.01%); soft palate (1, 1.01%); parapharynx (1, 1.01%); parotid/zygomatic arch (1, 1.01%); nasal cavity and sinuses (5, 5.05%); thyroid region (1, 1.01%); mandible (4, 4.04%); orbit (1, 1.01%); tongue (1, 1.01%); buccal mucosa (3, 3.03%); submandibular region (1, 1.01%); paralaryngeal region (1, 1.01%); retromolar triangle (1, 1.01%); supraclavicular region (2, 2.02%); infratemporal fossa (1, 1.01%); brain lobe (1, 1.01%); maxillary bone (1, 1.01%); gingiva (2, 2.02%); site not specified (28, 28.28%).
Clinical Presentation	Slow-growing mass (typical feature): 14 cases (14.14%); other presentations (painless swelling, subcutaneous masses, congestion, indurated mass, etc.): 85 cases (85.86%).
Surgical Treatment	Local excision/excisional biopsy: 28 cases (28.28%); wide local excision, subtotal resections, or craniectomy as required in other cases.
Mitotic Activity	No mitotic activity detected in 23 cases (23.23%); low mitotic index (≤2 mitoses/10 HPFs) in 26 cases (26.26%); mitotic data not reported in 50 cases (50.51%).
Atypia	Atypia absent in 31 cases (31.31%); atypia present (mild to moderate) in 20 cases (20.20%); atypia not reported in 48 cases (48.48%).
Recurrence	No recurrence documented in 31 cases (31.31%); recurrence documented in 9 cases (9.09%); recurrence status not reported in 59 cases (59.60%).
Metastasis	No metastasis observed in 40 cases (40.40%); metastasis documented in 2 cases (2.02%); metastasis status not reported in 57 cases (57.58%).
Follow-up Duration (years)	Range: 6 months to 33 years; mean: 6.21; SD: 3.50
Adjuvant Therapy	Adjuvant therapy used in 15 cases (15.15%); no adjuvant therapy in 84 cases (84.85%).

## Data Availability

No datasets were generated or analyzed during the current study.

## Supplementary Materials

The following supporting information can be downloaded at: https://www.mdpi.com/article/10.3390/cancers17091508/s1, File S1: PRISMA 2020 Checklist.
